# Quaternized Chitosan-Based Anion Exchange Membrane Composited with Quaternized Poly(vinylbenzyl chloride)/Polysulfone Blend

**DOI:** 10.3390/polym12112714

**Published:** 2020-11-17

**Authors:** Le Thi Tuyet Nhung, In Yea Kim, Young Soo Yoon

**Affiliations:** Department of Materials Science and Engineering, Gachon University, Bokjeong-dong, Seongnam-si 1342, Gyeonggi-do, Korea; letuyetnhung155@gmail.com (L.T.T.N.); gorl7484@gmail.com (I.Y.K.)

**Keywords:** anion exchange membrane, quaternized polymer reaction, crosslinking polymer backbone

## Abstract

An efficient and effective process for the production of high-performance anion exchange membranes (AEMs) is necessary for the commercial application of fuel cells. Therefore, in this study, quaternized poly vinylbenzyl chloride (QVBC) and polysulfone were composited with glycidyltrimethylammonium-chloride-quaternized chitosan (QCS) at different ratios (viz., 1 wt %, 5 wt %, and 10 wt %). The structure and morphology of the membranes were characterized by Fourier transform infrared spectroscopy and scanning electron microscopy, respectively. Further, the water uptake, swelling ratio, and ionic conductivities of the composite membrane at different wt % of QCS were evaluated. The membrane with 5% QCS exhibited an ionic conductivity of 49.6 mS/cm and 130 mS/cm at 25 °C and 70 °C, respectively.

## 1. Introduction

Recently, owing to the depletion of petroleum reserves and rising global warming, significant efforts have been devoted to the search for alternative sustainable energy sources [1,2,3,4,5,6,7]. In this regard, fuel cells have garnered significant interest owing to their high energy conversion efficiency, absence of environmentally toxic byproducts, and availability of wide fuel sources. Considering their manifold advantages, fuel cells have been utilized in stationary and mobile applications [8]. Among the low-temperature fuel cells, proton exchange membrane fuel cells (PEMFC) and anion exchange membrane fuel cells (AEMFCs) are of particular interest. Nevertheless, AEMFCs have several advantages over PEMFC, such as cost effectiveness and faster cathode reaction kinetics. Namely, PEMFCs utilize platinum group metals as catalysts, which increase the cost-to-performance ratio [9,10]. In contrast, AEMFCs that use non-noble metals, such as catalysts, and display faster reaction kinetics are economical from a commercial perspective [11,12]. In addition, fuel sources such as urea, ammonia, and biogas, which are more advantageous than other fuels, can be used in AEMFC [13,14]. In AEMFCs, ionic conduction is carried out by an anion exchange membrane (AEM) placed between the two electrodes. The ion-conducting groups are generally cationic functional groups grafted on polymer backbone chains [15,16,17,18]. However, AEMs generally display low ionic conductivity and stability compared to those of proton exchange membranes, which results in poor fuel-cell performance. Therefore, it is imperative to enhance the ionic conductivity and stability of AEMs. Ionic conductivity can be improved by increasing the gravimetric charge density. However, an increase in the ionic exchange capacity (IEC) leads to excessive swelling of the AEMs, which in turn leads to a decrease in fuel-cell performance. Anion exchange membranes were synthesized via the following routes: (i) radiation-grafting and quaternization of the polymer, (ii) polymer blended with alkali, (iii) pyridinium base-type polymer, and (iv) chloromethylation followed by the quaternization of polymer [14,19]. Another way to develop AEM is the incorporation of fillers into polymers by forming composites. For instance, the incorporation of QPVA/silica [20], Poly sunlfone(PSF)Al_2_O_3_ nanoparticles [21], and graphene oxide crosslinked poly(phenylene oxide) [22]. The composite AEMs did not only exhibit high ionic conductivity but also demonstrated excellent chemical stability, which is worthy of further investigation from a commercial perspective.

Therefore, this study proposed a directly quaternized process to synthesize a polymer membrane without the use of chlorine functional groups, which might potentially be carcinogens. A superior approach to avoid chlorination involves taking advantage of a polymer containing chloride groups. Consequently, poly(vinylbenzyl chloride) (PVBC) was chosen to introduce quaternary ammonium (QA) into polymer backbone chains. QA has been thoroughly researched because of its highly stable cations. However, its conductivity and chemical stability need to be enhanced [16,23,24]. A new approach to improve stability is by blending QVBC with polysulfone as a reinforcing matrix substrate. Polysulfone is known to have good chemical and mechanical stability, which encourages us to investigate it as a reinforcing matrix in AEM applications.

Quaternized PVBC mixed with quaternized CTS(Chitosan) was used as an anion exchange membrane to obtain mechanical-chemical stability and enhance conductivity. Chitosan is a natural macromolecule polymer, which is biodegradable, low-toxic, and inexpensive [25,26,27,28]. CTS has been studied as a polymer membrane in fuel cell systems owing to its high mechanical strength and impressive film-forming capability [28]. Meanwhile, the CTS membrane exhibited relatively low conductivity; therefore, (2,3-expoxylpropyl) trimethylammonium chloride (EPTMAC) was used to modify the CTS. Consequently, a CTS quaternary ammonium salt was formed, namely: 2-hydroxylpropyl trimethylammonium chloride chitosan (QCS) [24,27,29]. EPTMAC spontaneously reacted with the amino groups in CTS. The introduction of EPTMAC may provide more conductive anions (OH^−^). Although QCS is blended with the membrane, its exchangeable groups can increase the ion conductivity of the composite. This occurs because high ionic conductivity requires a high density of quaternary ammonium groups.

**Figure 1:**
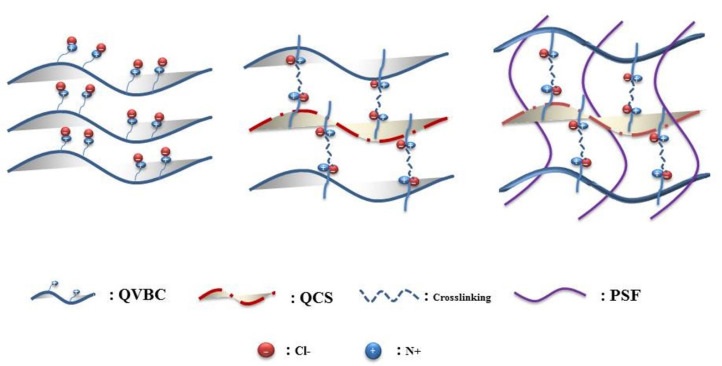
Schematics of membrane fabrication.

In this study, a novel composite membrane was synthesized using a simple method that involved the blending of functionalized chitosan/polyvinyl benzyl chloride and polysulfone as a filler to balance the membrane swelling, which in turn controls the membrane dimensions. It is believed that incorporating QCS into QVBC will not only lead to increased ionic groups, but also enhance ionic conductivity. The membrane’s formation was showed through a schematics in Figure 1.

## 2. Materials and Methods

### 2.1. Materials

Poly vinylbenzyl chloride (PVBC; 60/40 mixture of 3- and 4-isomers), N, N-dimethyl ethanol amine (DMEA), N-methyl-2-pyrrolidone (NMP), chitosan (CTS) (>75% deacetylated), and glycidyltrimethylammonium chloride (EPTMAC, ≥90%) were purchased from Sigma Aldrich (Gyeonggi-do, Korea). All reagents were of analytical grade and used as obtained without further purification.

### 2.2. Experimental

#### 2.2.1. Quaternized Chitosan

The quaternization of chitosan was carried out by following a report published elsewhere [26,28,29]. Briefly, deacetylated chitosan (6 g) was dispersed in 60 mL of deionized (DI) water at 85 °C by stirring vigorously. Then, EPTMAC (21.3 mL) was added slowly to the above dispersion. The reaction was allowed to stir for 10 h at 85 °C under an inner atmosphere, after which the whole reaction content was poured into 200 mL of cold acetone under stirring conditions and kept overnight at 4 °C in the refrigerator. Thereafter, acetone was decanted and the precipitate was dispersed in methanol (100 mL). A slurry-like material was formed, which was repeatedly washed with ethanol and acetone mixture in a ratio of 4:1 (250 mL). The white product obtained was repeatedly washed with hot EtOH using vacuum filtration to remove any impurities. Finally, the material was dried at 40 °C overnight to obtain quaternized chitosan [27,30,31].

**Figure 2:**
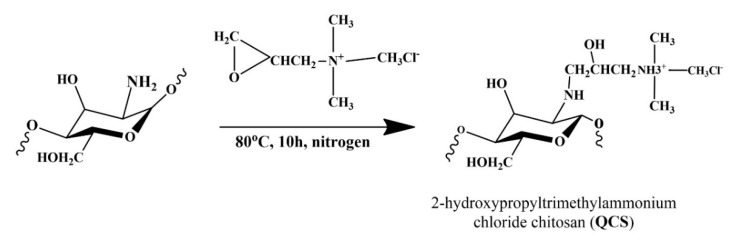
Synthesis of quaternized chitosan (QCS).

QCS was selected to synthesize the polymer membrane, the reaction was showed in the Figure 2. It was blended with QVBC to increase the performance of the fuel cell. In this study, crosslinked QVBC and QCS composite membranes were prepared, and their characteristics were investigated. In neutral aqueous conditions, the hydroxyl groups of chitosan were not nucleophilic enough to induce the ring opening of EPTMAC, whereas the amino group of chitosan was nucleophilic enough to do that (Roberts 1992). Hence, this reaction mostly took place at –NH_2_ in C–2. The ring-opening reaction occurred on EPTMAC, which substituted –H at –NH_2_ to add quaternary ammonium groups onto the chitosan backbone [32].

#### 2.2.2. Synthesis of Positively Quaternized Vinyl Benzyl Chloride (QVBC)

**Figure 3:**
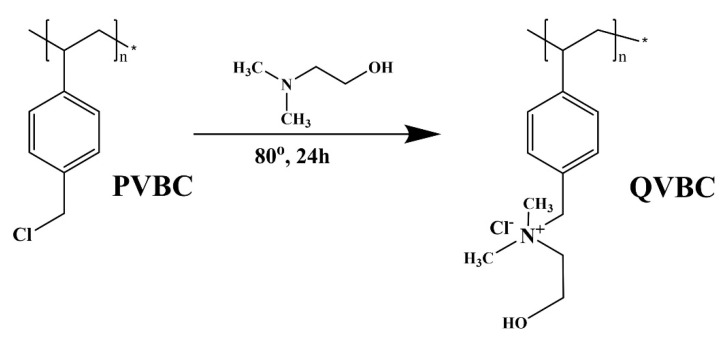
Synthesis of positively quaternized vinyl benzyl chloride (QVBC).

The quaternization of poly vinylbenzyl chloride is illustrated in Figure 3. In a four-neck round-bottom equipped glass, 2 g of PVBC was dissolved in 10 mL of NMP, and 0.5 mL of N, N-dimethyl ethanol amine under the inner atmosphere. The reaction was carried out at 80 °C for 24 h and then precipitated in 100 mL diethyl ether, and then it was washed at least twice with diethyl ether to remove the unreacted substances from quaternization. The product was dried in a vacuum oven at 40 °C for 24 h.

#### 2.2.3. Membrane Preparation

**Figure 4:**
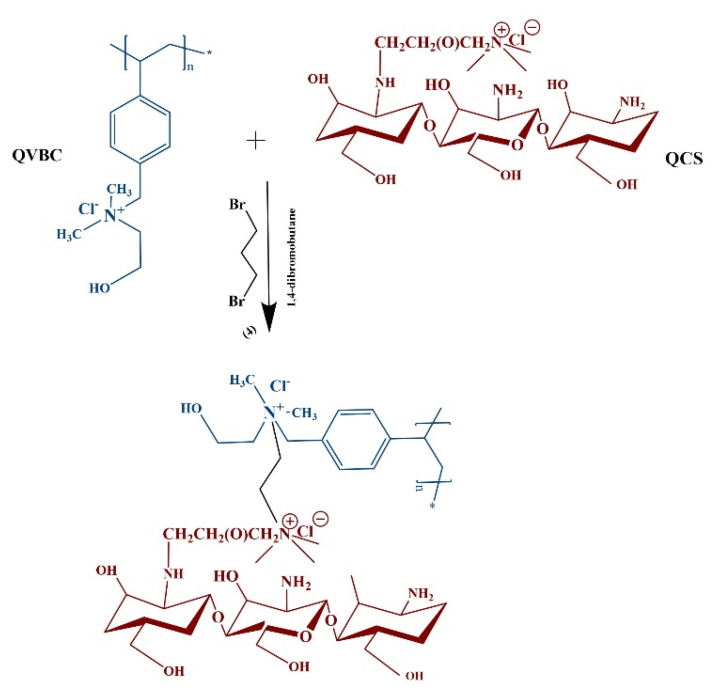
Crosslinking and membrane formation.

A schematic representation of the composite membrane is shown in Figure 4. The quaternized polymer QVBC was first dissolved in NMP solvent and stirred at an ambient temperature until a homogeneous solution was formed; subsequently, QCS (0, 1, 2, 5, and 10 wt %) was dissolved in acetic acid (2%). The composite membrane was crosslinked with 1,4-dibromobutane, and the mixture was stirred for 30 min at 60 °C. To obtain the crosslinked membranes, the solution was poured into a Teflon dish and dried in a vacuum oven at 50 °C for 24 h. It was peeled off and then immersed in 1 M KOH solution for one day to convert the Cl^−^ form of the membrane into OH^−^ form [26,33,34,35,36,37].

## 3. Analysis/Characterization

### 3.1. Fourier Transform Infrared (FTIR) Characterization

After a dehydration process at 40 °C for 24 h in a vacuum oven, the FTIR spectra of the membrane samples were obtained using a Bruker Vertex 70 FT-IR spectrometer (Bruker, Billercia, MA, USA) in the wavenumber range of 500–4000 cm^−1^ in transmittance mode.

### 3.2. Nuclear Magnetic Resonance (NMR) Spectra

The ^1^H-NMR spectra were obtained using a high-resolution NMR spectrometer (Bruker BioSpin GmbH, Ettlingen, Germany) at room temperature and deuterated dimethyl sulfoxide (DMSO-*d*^6^) as the solvent.

### 3.3. Thermal Stability of the Membranes

Thermogravimetric analysis (TGA) was performed to analyze the thermal properties of the membranes using a thermogravimetric analyzer (SDT Q600 V20.9 Build 20, Artisan, IL, USA) at a heating rate of 10 °C/min over a wide temperature range from ambient environment to 500 °C under a nitrogen atmosphere.

### 3.4. SEM Characterization of the Membranes

The membrane morphology was observed evaluated with a scanning electron microscope (FE-SEM, JEOL JSM-7500F, JEOL, Tokyo, Japan).

### 3.5. Water Uptake (WU), Swelling Ratio (SR) of the Membrane

The WU of the membrane was measured by the weight difference between the wet membranes (M_wet_) and the dry membrane (M_dry_).

After determining M_dry_, the membranes were soaked with deionized (DI) water for 24 h at room temperature to determine the variation in weight and the dimensions of the wet membranes.

Before gauging M_wet_, the soaked membrane was quickly wiped with a tissue. The WU of the membrane was evaluated using the following equation:

$$\mathrm{WU}\;(\%)\;=\frac{M_{\mathrm{wet}}-M_{\mathrm{dry}}}{M_{\mathrm{dry}}}\times 100\%$$

The change in dimensions of the membrane supports in calculating SR. The dimensions of the soaked membrane were collected after removing the excess water with tissue. The SR of the membrane was defined using the following equations:

$$\mathrm{SR}\;(\%)=\frac{L_{\mathrm{wet}}-L_{\mathrm{dry}}}{L_{\mathrm{dry}}}\times 100$$

where $L_{\mathrm{wet}}$ and $L_{\mathrm{dry}}$ are the length of the soaked and dried membranes, respectively.

### 3.6. Ionic Exchange Capacity (IEC) of Membranes

The IEC values of the membranes were measured using the classical titration method. First, the membranes were cut into pieces and soaked in NaOH solutions (0.1 M) for 24 h. Next, the pH solution was increased to 7 by using 0.1 M HCl solution, whereas the indicator was phenolphthalein. Then, the membranes were rinsed with DI water several times and dried under vacuum conditions at 50 °C for 24 h. The IEC (mmol/g) of the membranes was evaluated using the following equation:

$$\mathrm{IEC}\;(\mathrm{mmol}/g)=\frac{\left(V_{b}-V_{r}\right)\times C_{NaOH}}{M_{\mathrm{dry}}}$$

where *V_b_* and *V_r_* are the amount of NaOH before and after titration, respectively; *C_NaOH_* represents the concentration of the NaOH solution.

### 3.7. Ionic Conductivity

The Nyquist plots of the exchange membranes were obtained by a two-probe electrochemical AC impedance method. The membranes were washed with deionized water before clamping them between the two copper electrodes using a resistance. The inert atmosphere was conserved by using N_2_ throughout the impedance measurement. The amplitude was 50 mV, for the frequency varied from 7 MHz to 100 kHz and back. The ionic conductivity was estimated using the following equation:

$$\delta \;=\;\frac{L}{\left(R\times A\right)}$$

where L (cm), R (ohm), and A (cm^2^) represent the separation between the two electrodes, resistance obtained from the Nyquist plot, and surface area, respectively.

## 4. Results and Discussion

### 4.1. Fourier Transform Infrared (FTIR) Characterization

**Figure 5:**
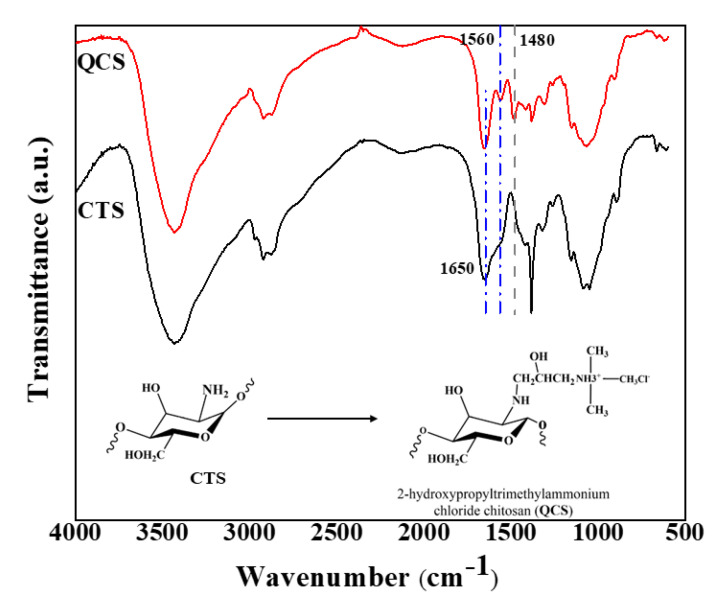
FTIR spectra of chitosan and quaternized chitosan.

FT-IR was used to confirm the success of the quaternization. Figure 5 illustrates the FT-IR spectra of chitosan and quaternized chitosan. For chitosan (CTS), the peaks appear at 3289, 2924, 1650, and 1072 cm^−1^ [38]. The peak at 3400–3500 cm^−1^ represents the characteristic absorbance of –NH_2_ and –OH. The weak peak at 2924 cm^−1^ shows the stretching vibration of –CH [39,40]. The band at 1072 cm^−1^ can be attributed to the stretching vibration of C–O.

The FT-IR spectrum of QCS verifies the introduction of a quaternary ammonium salt group on the chitosan backbone. The peak at 1480 cm^−1^ is ascribed to the C–H asymmetric bending vibration of the trimethylammonium group, thus confirming the existence of quaternary ammonium salt [26,31,32,39]. It should also be noted that the N–H bending (1560 cm^−1^) of the primary amine vanished because of the transformation of the primary amine into the secondary amine (aliphatic). The intensity ratio between peaks at 1560 cm^−1^ (NH_2_) and 1650 cm^−1^ (C=O) was 0.74 for CTS (Figure 5). Meanwhile, the aforementioned ratio decreased to 0.42 for QCS, because the primary amine group vanished and the C=O group (in CTS’s chains) remained unchanged after the quaternization.

In addition, the spectrum exhibits a broad band at approximately 3450 cm^−1^, which became wider than that of chitosan owing to the increased number of hydroxyl groups. This result illustrates that the substituted reaction mostly took place at NH_2_ of the chitosan.

**Figure 6:**
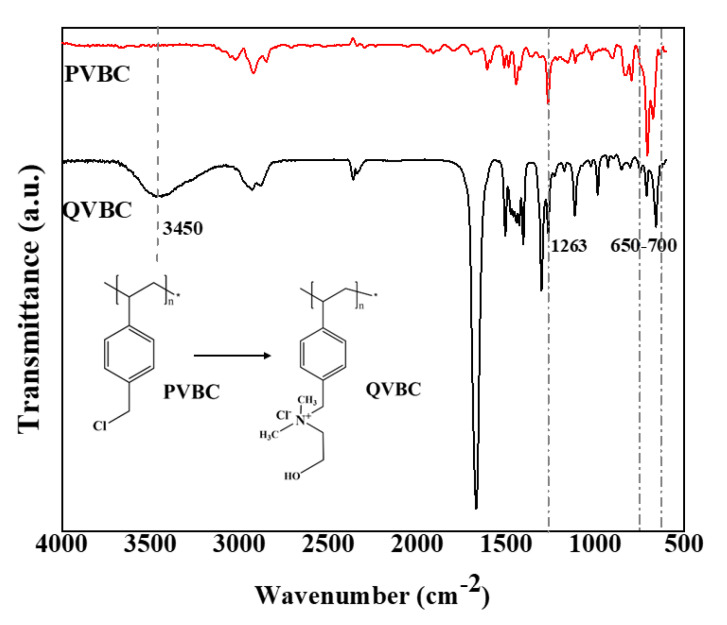
FTIR spectra of PVBC (poly vinylbenzyl chloride) and positively quaternized poly vinylbenzyl chloride (QVBC).

Figure 6 illustrates the FT-IR spectra of PVBC and QVBC, respectively. From the PVBC spectrum, the peak, which appeared in the region of 650–705 cm^−1^ can be ascribed to the C–Cl stretching, whereas that at 1263 cm^−1^ is assigned to CH_2_Cl wagging vibrations. The intensity ratio between peaks at 650–700 cm^−1^ (C–Cl) and 2924 cm^−1^ (C–H) of PVBC (3.7) was higher than that of QVBC (2.2). It was because the quaternization consumed the C–Cl group in PVBC to result QVBC.

In contrast, the C–Cl stretching and CH_2_Cl wagging of PVBC thoroughly vanished in the QVBC’s spectrum, which divulged a complete quaternization of the CH_2_Cl group after the amination process. Additionally, a new peak appeared at 1476 cm^−1^ and was attributed to the asymmetric stretching and bending of C–H of the –N(CH_3_)_2_ quaternary ammonium groups [40,41]. Furthermore, the quaternization also delivered the O–H group to QVBC, so a new peak appeared at 3450 cm^−1^. Accordingly, it is evident that the quaternization reaction was successful.

### 4.2. Nuclear Magnetic Resonance (NMR) Spectra

**Figure 7:**
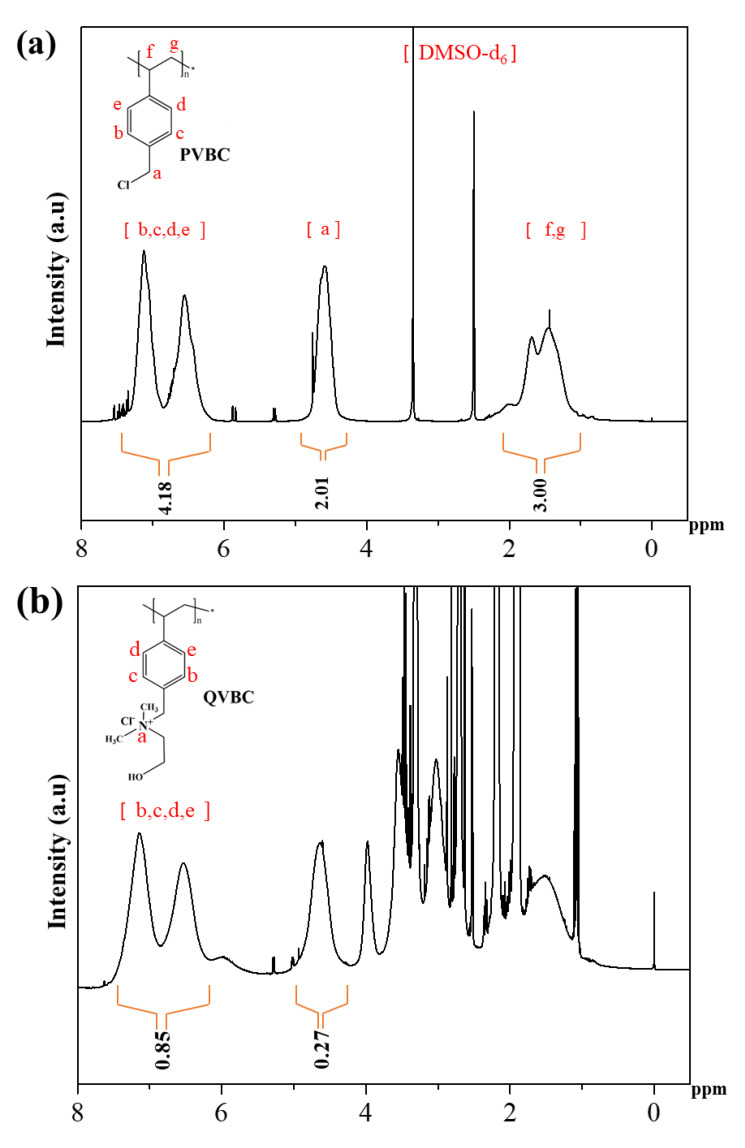
(**a**) ^1^H-NMR spectra of PVBC and (**b**) QVBC-quaternized PVBC.

Figure 7 displays the ^1^H-NMR spectra of PVBC and QVBC. The NMR spectra illustrated in Figure 7a indicate the presence of aliphatic-proton peaks of –CH_2_ and –CH groups of PVBC at 1 to 2 ppm, while the aromatic ring protons are observed at 6.5 and 7.2 ppm. The peak at approximately 4.6 ppm is determined as the CH_2_Cl group. Besides, DMSO-*d*_6_ and its moisture caused peaks approximately at 2.5 and 3.3 ppm, respectively.

Figure 7a shows that the area ratio between proton peaks at 4.3 to 4.9 ppm (2.01) and at 6.5 to 7.2 ppm (4.18) was 0.48, which is highly consistent with the theoretical ratio between protons in CH_2_Cl groups and rings in PVBC (0.5). Figure 7b implies that the area ratio between protons of the CH_2_Cl peak (0.27) and that of the rings (0.58) was only 0.32 for QVBC. As the quaternization caused a depletion of CH_2_Cl groups, QVBC was lower than PVBC at the proton ratio of CH_2_Cl groups to rings. Accordingly, the decrease in the aforementioned proton ratio divulged a quaternization yield of 33.3% approximately. Further, the new peak observed at 3.72 ppm in Figure 7b corresponds to the ammonium quaternary group. These results confirm that the quaternization of the PVBC with DMEA was successful.

### 4.3. Thermomechanical Stability of Membranes

**Figure 8:**
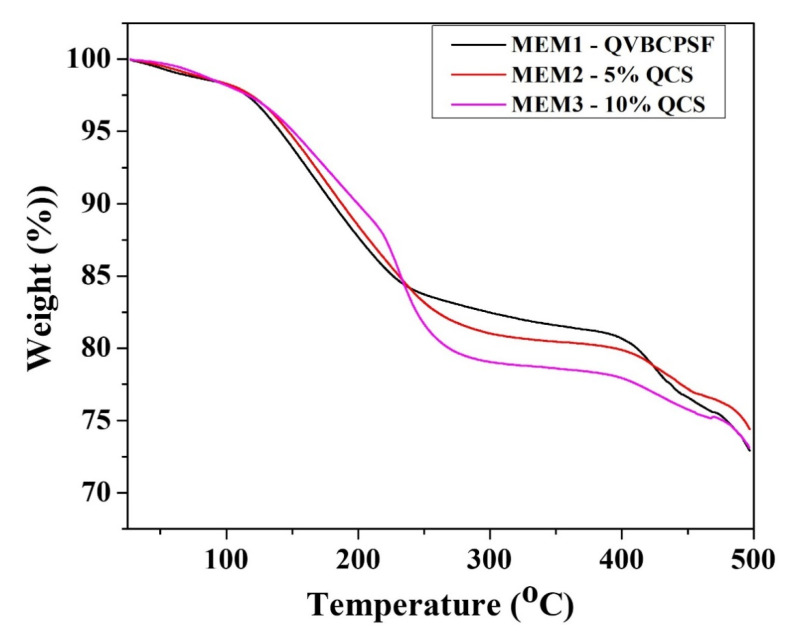
TGA curves of different ratio membrane.

The thermal behavior of the blended QVBC, PSF, and QCS membranes were analyzed by TGA, and the results are presented in Figure 8. Until 300 °C, TGA curves of MEM1-0% QCS and MEM2-5% QCS membranes consisted of two main weight loss (below 110 and above 110 °C). At a significant amount of QCS, the TGA curves of MEM3 membrane additionally included a substantial loss stage beginning at 220 °C because of the lesser stability of QSC [42]. The initial weight loss for temperatures up to 110 °C is related the expulsion of water molecules, which can be elucidated by the absorption of moisture by the membranes because of the presence of hydrophilic ionic groups. The second step involves the decomposition of quaternary ammonium groups and cleavage of the QCS main chain because the thermal temperature of QCS is up to 230 °C, as reported previously [41]. The weight loss corresponding to the last stage is due to deformation of the crosslinking network and polymer backbone [40,43]. It can be observed that PVBC exhibits excellent thermomechanical stability up to 350 °C. The weight loss of the membrane at 350 °C, owing to the decomposition of the CH_2_-Cl groups and the main chain above 450 °C [24,34]. It should be noted that the temperature required for the degradation of MEM3-10% QCS is relatively lower than that necessitated for the others. This shows that the QCS content affects the thermoplastic behavior of the anion exchange membrane.

In summary, the blend membrane QVBC/PSF/QCS-OH reveals good thermal stability below 70 °C, which is suitable for application in low-temperature fuel cells.

### 4.4. SEM Characterization of Membranes

**Figure 9:**
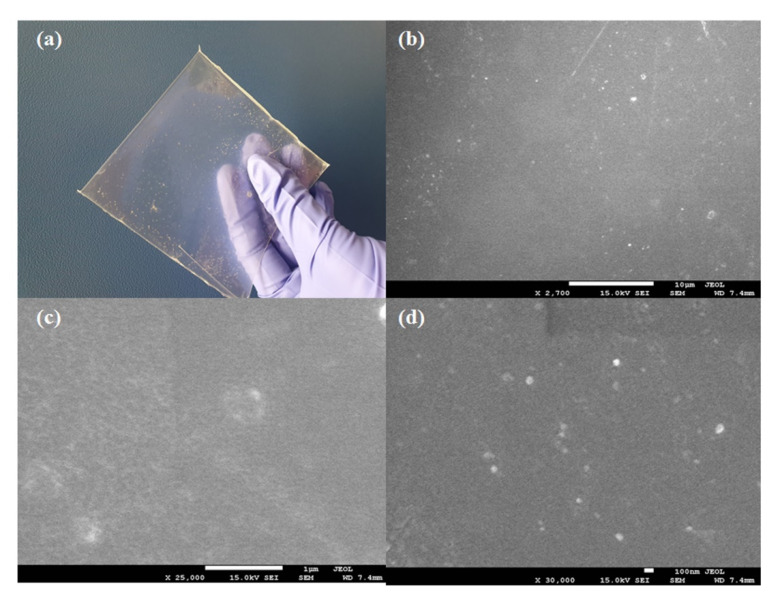
(**a**) Optical image; SEM images of (**b**) MEM1-0% QCS, (**c**) MEM2-5% QCS, and (**d**) MEM3-10% QCS.

The optical image and SEM image of the QVBC/PSF/QCS membrane are illustrated in Figure 9. The optical image in Figure 9a shows that the QVBC/PSF/QCS membrane was transparent, indicating no visible agglomeration of the chitosan particles. Figure 9b–d shows that all the prepared membranes were uniform and continuous. Because the crosslinking reaction joins the components together via chemical bonds, the obtained membrane is compact and homogeneous.

### 4.5. Properties of Composite Membrane

The IEC, swelling ratio, and water uptake of the blended membranes are listed in Table 1. The IEC is an essential parameter of the ion exchange membrane and related to the amount of exchangeable ionic within the membrane, which is strongly correlated with the ionic conductivity and water absorption of the membrane. The IEC of the composite membrane increased from 0.9 to 2.24 mmol/g, and the pristine QVBC/PSF exhibited an IEC of 0.9 mmol/g. As expected, the water uptake and swelling increased with the increase in temperature and IEC. At higher IEC values, water absorption was higher, which may indicate that the amount of QA groups in the blending polymers directly augments the localized water content.

**Figure 10:**
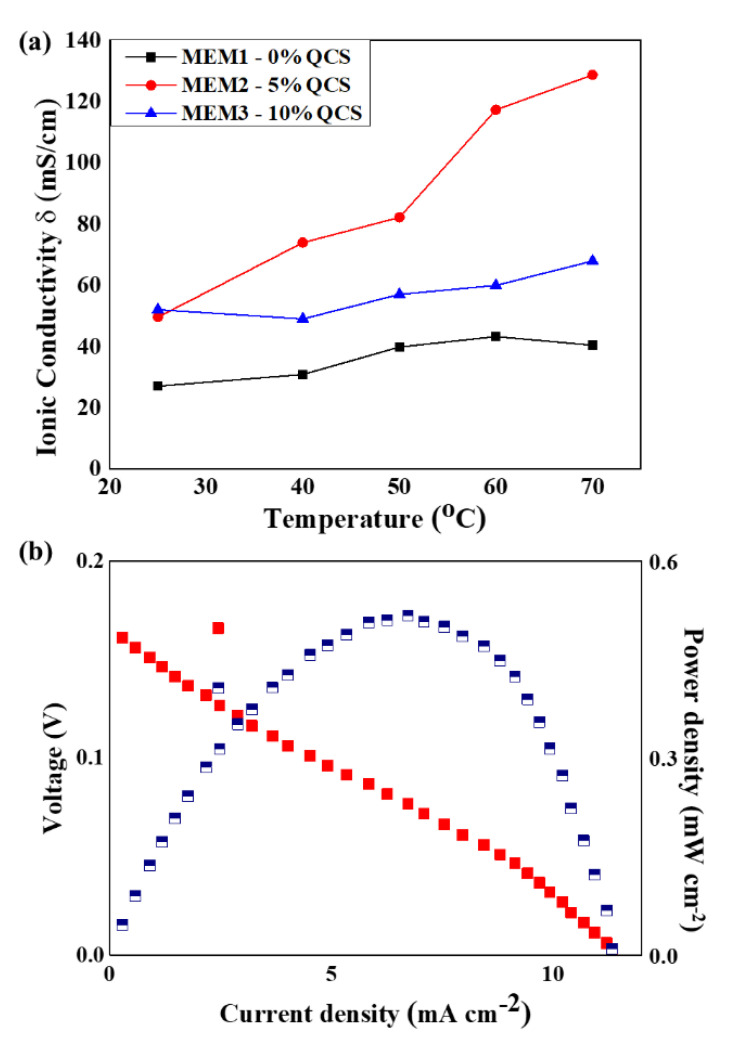
(**a**) Ionic conductivity with variation in temperatures for different membranes; (**b**) urea/O2 test result for MEM2, combined fuel cell I-V (red cubes) and power density curve (blue cubes).

As mentioned above, the increase in water absorption is mainly because of an increase in the IEC value. Furthermore, the polar functional groups in chitosan are hydrophilic and increase the water uptake of the composite films. As the ionic movement relies on the water absorption, a higher water uptake may lead to better ionic conductivity. This is the major reason why QVBC/PSF (MEM1) exhibited the lowest ionic conductivity and other inferior properties, and is indicative of the fact that the number of cationic groups is less than QVBC/PSF/5%QCS (MEM2) and QVBC/PSF/10%QCS (MEM3). However, with an increase in temperature, MEM1 exhibited a gradual increase in its ionic conductivity with temperatures ranging from RT to 60 °C (Figure 10a). This can be explained by the temperature dependence of the ionic exchange, and the transfer of ionic ions, which is faster at high temperatures.

It can be seen that when water uptake increased, the dimension of the membranes changed, which was demonstrated via the measurement of the swelling ratio (Table 1). In contrast, excessive water leads to excessive swelling, thereby deteriorating the mechanical properties of the membrane. Generally, the existence of the QCS increases the deformation of the membrane. Namely, MEM2 and MEM3 membranes are higher at in-plane and through-plane swelling ratios than MEM1 membrane.

Figure 10b shows the dependencies of the cell’s voltage and power density on the current density. The cell delivered a maximum power density of 0.52 mW cm^−2^ at a current density of 6.7 mA cm^−2^.

The MEM2 cell is comparable with the cell made by G. Das et al. at the power density [44]. However, the low voltage may come from the catalyst’s unoptimized composition.

Table 1 shows the ionic conductivities of the blended membranes. During the membrane ionic conductivity measurements, a continuous N_2_ atmosphere was created in the conductivity cell to avoid any carbonate contamination. The pristine QVBC/PSF (MEM1) displayed the lowest ionic conductivity (27.04 mS/cm) among the other membranes at 25 °C. In the QVBC/PSF/5%QCS case, the ionic conductivity increased by approximately 1.8-fold compared to that of ordinary membrane, whereas QVBC/PSF/10%QCS (MEM3) exhibited an approximate 1.9-fold increase compared to that of pristine membrane (Table 1). The existence of two different phases formed of a clearer hydrophilic/hydrophobic phase separation, which results in well-connected ion channels that facilitate faster hydroxyl transport. As can be observed, ionic conductivity was proportional to the water content. Water content plays a crucial role in the formation and transportation of charge carriers and the development of connecting ionic domains. Nonetheless, higher water uptake in QVBC/PSF/10%QCS sample can decrease the carrier charges and lower ionic conductivity. As the existence of water influence to the conductivity of the membrane, there is usually an optimal water uptake to maximize the performance of the cell [45,46].

The dependence of the ionic conductivity on temperature was also investigated for the temperature range of 25–70 °C (Figure 10). At higher temperatures, conductivity was found to be significantly higher than that observed at room temperature. At 70 °C, the QVBC/PSF (MEM1) disclosed an ionic conductivity of approximately 40 mS/cm at, whereas the highest ionic conductivity of approximately 130 mS/cm was achieved by QVBC/PSF/5%QCS (MEM2) (Figure 10a). This demonstrates that hydroxyl ions can be quickly converted using efficient hydration ion channels. Therefore, the quaternized QVBC, which was highly compatible with QCS induced by crosslinking, resulted in well-separated ionic domains. A higher chitosan content might offer a “blocking effect” that limits the free ion transport and decreases conductivity. Additionally, G. Das et al. suggested that high water content may dilute the solute in the membrane and decrease the ionic conductivity [44]. As QCS increased WU, a QCS-based membrane could reach a maximum ionic conductivity at an optimized QCS. It seems like the optimized QCS content was 5%; therefore, MEM3 sample was lower at ionic conductivity than MEM2 sample at elevated temperatures.

The Table 2 was mentioned WU, IEC and IC from different membranes synthetic method for comparasion. PPO(Poly(phenylene oxide)) functionalized with quaternary groups via flexible heptyl spacer units was synthesized successfully using quaternization steps and straightforward bromoalkylation. The presence of a spacer tends to facilitate phase separation and promote ionic clustering, resulting in high ionic conductivity. In addition, it can avoid the efficient hydration of the QA groups and benzylic attachment, which is expected to enhance stability under alkaline solutions. When the IEC value was increased, the conductivity of PPO-7Q-1.8 increased to 85 mS/cm at 80 °C [43]. The other materials also exhibited high ionic conductivity with novel materials. Recently, numerous scientists have been interested in chitosan because of its properties; Das et al. [44] modified chitosan by employing methylation and subsequently crosslinking DMC(Dimethyl chitosan) with polymer chains to enhance the functional groups, which led to the formation of a significant number of hydroxyl ions. The DMC content decided the ionic conductivity of the membranes. The membranes exhibited good dimensional and stable membranes under hydrated conditions. The ionic conductivity of the membrane reached its peak with 2 wt % DMC, i.e., 54 mS/cm, and 94 mS/cm at 25 °C and 70 °C, respectively. In addition, cellulose was used to form the membrane for the first time and control the IEC and ionic conductivity of the anion exchange membrane, as observed in the literature [47]. The crosslinking of the polymer main chains and quaternized cellulose formed composites which was chemically and mechanically stable in an aqueous and alkaline environment. It was believed that the crosslinking of the polysulfone backbone and cellulose would benefit the AEM system by preventing excessive water uptake. By increasing the loading of quaternized chitosan, the ionic conductivity of the membrane increases 170% and achieve 74.23 mS/cm higher than that of QPSf/DBB without quaternized cellulose.

Generally, the amount of ions across a membrane decides the ionic conductivity. As graphene and sulfonated graphene destroyed crystallinity, sulfonated membrane increased in hydroxide conductivity and decrease of destruction. The corresponding values of the chitosan/PVA/sulfonated graphene membrane reached 0.0476 S/cm [48]. Similar to graphene, molybdenum sulfide (MoS_2_) nanomaterials dramatically enhanced the performance of the QPVA/chitosan composite membranes [49]. At an optimum MoS_2_ content 0.2 wt %, the ionic conductivity was 32 mS/cm. Furthermore, the MoS_2_-modified membrane was approximately 460% times greater at selectivity than the pristine one. In this study, the composite membrane QVBC/PSF/10%QCS showed highest ionic conductivity 130 mS/cm at the lower water uptake at 70 °C, that demonstrated the content of water was controlled well with 10% of QCS. The amount of functional groups on polymer chains decides the formation of well-connected hydrophilic channels. Due to the construction of continuous hydrophilic channels in the membrane, OH’s transport behavior can be significantly improved [50,51].

As the preparation of the desired membrane employed industrially available feedstock, the prepared membrane may be affordable for commercial AEMFCs. Nevertheless, a chitosan-based membrane should be carefully designed to limit the deformation of the membrane during operation.

## 5. Conclusions

A novel blending anion exchange membrane was successfully synthesized using a QVBC blend with quaternized QCS to enhance quaternary ammonium groups and increase the exchange of hydroxyl ions. Additionally, PSF is well known for its flexibility and stability; therefore, it has been used as a filler to improve the mechanical properties of the membrane. The membrane with a low PVBC intake is crisp in nature because of the presence of a high amounts of hydrophobic groups that render the polymer rigid. Therefore, QCS plays an important role in this study; QCS is not only cost-effective and environmentally friendly, but also highly hydrophilic, which helps increase the water uptake of the membrane. The membrane surface is extremely smooth with no defects or cracks. Furthermore, the membranes also exhibit excellent thermal stability with an onset degradation temperature that significantly exceeds 170 °C. The composite membrane not only improved the mechanical strength properties, but also the thermal characteristics.

## Figures and Tables

**Table 1:** Properties of membranes.

	Water Uptake (%), RT	Ion Exchange Capacity (mmol/g)	Ion Exchange Capacity (mmol/cm^3^)	Through-Plane Swelling Ratio (%) at RT	In-Plane Swelling Ratio (%) at RT	Ionic Conductivity (mS/cm) at RT
**QVBC/PSF 1–0% QCS**	39.4	0.9	0.96	20.5	6.38	27.04
**QVBC/PSF 2–5% QCS**	61.7	1.98	1.56	28.1	10.4	49.6
**QVBC/PSF–10% QCS**	66.7	2.24	1.75	38.5	8.1	52

**Table 2:** Comparison of IC obtained via different approaches.

Membrane	Modification	Water Uptake (%), RT	Ion Exchange Capacity (mmol/g)	Ionic Conductivity (S/cm)	References
**QVBC/PSF/10%QCS**	Crosslinking and quaternization	61.7	1.7	0.13 (70 °C)	This study
**PPO-QA**	Straightforward bromoalkylation and quaternization steps	65	1.8	0.085 (80 °C)	[43]
**QPSfDMC**	Crosslinking and quaternization	124	2.34	0.094 (70 °C)	[44]
**QPSf/QC**	Quaternized cellulose and crosslinking	80.47	2.71	0.128 (80 °C)	[47]
**PVA/Chitosan/Graphene (PCsG0.1)**	Sulfonated graphene	x	x	0.093 (80 °C)	[48]
**QPVA/Chitosannanoparticles (10%) (CQPVA-CL)**	Chitosan nanoparticles	89.5	x	0.032 (70 °C)	[49]
